# Evaluation of changes in objective visual quality based on tear film stability after SMILE surgery

**DOI:** 10.3389/fmed.2025.1538359

**Published:** 2025-04-25

**Authors:** Tao Liao, Lili Li, Diefeng Wei, Hejuan Mao, Yanyan Huang, Pengfei Lu, Dedong Zhong, Haiyan Lu, Huiyao Huang, Qi Chen

**Affiliations:** ^1^Visual Science and Optometry Center, The People's Hospital of Guangxi Zhuang Autonomous Region, Guangxi Key Laboratory of Eye Health, Guangxi Health Commission Key Laboratory of Ophthalmology and Related Systemic Diseases Artificial Intelligence Screening Technology, Institute of Ophthalmic Diseases, Guangxi Academy of Medical Sciences, Nanning, China; ^2^Guangxi Medical University, Nanning, China

**Keywords:** SMILE, objective visual quality, OQAS II, dry eye, tear film stability

## Abstract

**Purpose:**

This study aimed to evaluate changes in visual quality among myopic patients with varying tear film stability after small incision lenticule extraction (SMILE) using the Optical Quality Analysis System (OQAS II).

**Methods:**

This prospective study analyzed 141 patients who underwent SMILE surgery, selecting the right eye of each patient for analysis. Objective visual quality and tear film stability were assessed using the OQAS II preoperatively and at 1 week, 1 month, and 3 months postoperatively. Refractive error and uncorrected visual acuity were measured at the same time points. At the 1-week follow-up, all patients completed a visual quality questionnaire and underwent tear film break-up time (TBUT) measurement. These assessments were conducted to evaluate the correlation between subjective visual perception and objective visual quality, as well as to examine the relationship between the two methods of tear film evaluation.

**Results:**

No significant differences in UDVA or postoperative spherical and equivalent spherical values were observed between groups at any postoperative time point (*p* > 0.05). At 1 week and 1 month post-surgery, both groups exhibited elevated Objective Scatter Index (OSI) and Mean OSI values, while OV100%, OV20%, OV9%, MTF cutoff, and Strehl ratio (SR) decreased compared to preoperative levels (all *p* < 0.05). By 3 months post-surgery, all objective visual quality parameters in the tear film stability group returned to preoperative levels (*p* > 0.05). In contrast, in the instability group, only SR remained unchanged, while other parameters significantly differed from baseline (*p* < 0.05). Positive correlations were found between OSI values and both foggy vision and glare at 1 week postoperatively in both groups. TF-OSI values positively correlated with blinking frequency and visual fluctuations. OV100% values at all preoperative and postoperative time points did not effectively predict subjective visual acuity.

**Conclusion:**

Tear film instability negatively impacts visual quality recovery and prolongs corneal healing time after SMILE surgery. However, it does not cause short-term refractive regression. OV100% shows limited predictive ability for visual acuity. A significant correlation exists between objective visual quality and subjective perception. The OQAS II system is a valuable tool for assessing tear film stability and objective visual quality in refractive surgery patients.

## Introduction

Refractive errors are a leading cause of correctable vision deficits worldwide (1). With the increasing global prevalence of myopia (2) and advancements in modern medical technology, small incision lenticule extraction surgery has become a common and emerging refractive surgical procedure. SMILE surgery uses a femtosecond laser to create a microlens within the corneal stroma and removes it through a small incision. This technique is notable for eliminating the need for an open corneal flap (3), thereby reducing the incidence of flap-related complications. In recent years, numerous studies have demonstrated that the SMILE procedure offers significant advantages, including improved visual acuity, enhanced refractive stability, and a reduced incidence of surgical complications. Compared to previous refractive surgical techniques, SMILE is characterized by smaller, safer incisions and more accurate correction of refractive errors (4, 5). The primary goal of refractive correction is not only to restore clear vision but also to enhance visual comfort and durability. Therefore, special attention must be given to the overall visual quality of patients after surgery.

Dry eye is a common complication following refractive surgeries (4), including SMILE. The suction device used during SMILE not only affects the corneal layers but also damages the conjunctival goblet cells. This damage may compromise the corneal–conjunctival epithelium, leading to mucin deficiency and tear film instability, thereby contributing to the development of dry eye (6). Zou et al. analyzed the relationship between tear film stability and dry eye, concluding that a shorter tear film break-up time is the most common clinical sign of dry eye (7). Tan et al. investigated the impact of dry eye on functional vision and quality of life, demonstrating that dry eye significantly reduces functional vision and negatively affects quality of life (8). Previous studies have used the OQAS II visual quality analysis system and other tools to assess the effect of different eye drop concentrations and compositions on objective visual quality and phase difference in dry eye patients (9–11). However, the specific impact of tear film stability on visual quality after refractive surgery has not been thoroughly analyzed in existing studies. Therefore, this study aims to evaluate the influence of tear film stability on both objective visual quality and subjective perception in patients undergoing refractive surgery.

## Methods

### Patients

This prospective cohort study enrolled 141 patients (141 eyes) who underwent SMILE at the Visual Science and Optometry Center of the People’s Hospital of Guangxi Zhuang Autonomous Region between October 2022 and April 2024. The study was approved by the Ethics Committee of the People’s Hospital of Guangxi Zhuang Autonomous Region (No. IIT-2022-27) and adhered to the Declaration of Helsinki (2013 revision). Written informed consent was obtained from all participants. We excluded all patients with conditions other than refractive errors, including meibomian gland dysfunction, glaucoma, cataracts, as well as ocular or other systemic diseases. All patients underwent objective visual quality assessment using the OQAS II system 1 week postoperatively. Based on the postoperative tear dynamic scattering index (TF-OSI), defined as the mean OSI minus OSI, patients were categorized into two groups: the tear film stabilization group (Group A) with a TF-OSI value less than 1.2 and the tear film instability group (Group B) with a TF-OSI value of 1.2 or greater. The threshold setting follows the OQAS II standard operating manual, which states that a TF-OSI of ≥ 1.2 can be considered indicative of a dry eye patient. In addition, research by Yu Chen et al. (12) indicates that a TF-OSI > 1.13 has a diagnostic value for dry eye. For data collection convenience, we adopted a TF-OSI of 1.2 as the grouping criterion. To analyze the correlation between subjective visual perception and objective visual quality, the primary groups were subdivided based on the Tear Film Scattering Index (OSI) at 1 week postoperatively. Group A was subdivided into two subgroups: Group A1 (OSI ≥ 2) and Group A2 (OSI < 2). Similarly, Group B was divided into Group B1 (OSI ≥ 2) and Group B2 (OSI < 2).

### Surgical procedure

All procedures were performed by a single, highly experienced surgeon using the VISUMAX 500 laser system (Carl Zeiss Meditec AG, Jena, Germany) for small incision lenticule extraction (SMILE). This laser device operates at a frequency of 500 kHz, with each pulse delivering 130 nJ of energy. The dimensions of the lenticule were individually customized, with diameters ranging from 6.0 to 6.5 mm. The cap’s diameter was set between 7.0 and 7.5 mm, and its thickness was adjusted from 110 to 120 μm. Spiral-patterned incisions were created on both the front and back surfaces. A 2.0-mm wide incision was made at a 120°angle.

All patients strictly adhered to medication guidelines both before and after surgery. The specific medications were administered as follows: Preoperative regimen: Beginning 3 days prior to surgery, all subjects were prescribed Levofloxacin Eye Drops (Cravit, Santen Pharmaceutical Co., Ltd., Japan) and 0.3% Sodium Hyaluronate Eye Drops (Hialid, Santen Pharmaceutical Co., Ltd., Japan), each to be applied four times daily. Postoperative medication: For the first week following surgery, subjects were instructed to use Tobramycin and Dexamethasone Eye Drops (Tobradex, ALCON Laboratories, Belgium) four times daily, 0.3% Sodium Hyaluronate Eye Drops (Hialid, Santen Pharmaceutical Co., Ltd., Japan) four times daily, and Deproteinized Calf Blood Extract Eye Gel (Shenyang Xingqi Eye Pharmaceutical Co., Ltd., China) twice daily. From 1 week to 1 month post-surgery, patients discontinued the Tobramycin and Dexamethasone Eye Drops, replacing them with Fluticasone Eye Drops while maintaining all other medications. After the 1-month mark, all medications were discontinued.

### Preoperative and postoperative assessment

All assessments were performed by the same examiner. Prior to surgery, all patients underwent a comprehensive ophthalmic examination. The assessment included a detailed slit-lamp biomicroscopy to evaluate the anterior segment of the eye. Tear film stability was assessed by measuring the tear breakup time (TBUT) using a non-invasive method with the Oculus Keratograph 5 M (K5, Oculus GmbH, Wetzlar, Germany). The Optical Quality Analysis System II (OQAS II, Visiometrics S.L., Terrassa, Spain) was utilized to measure the following parameters: modulation transfer function cutoff frequency (MTF cutoff), objective scatter index (OSI), mean objective scatter index (Mean OSI), predicted visual acuities at 100% contrast (OV100%), 20% contrast (OV20%), and 9% contrast (OV9%), as well as Strehl ratio (SR). Intraocular pressure and anterior segment analysis (Pentacam HR; Oculus, Germany) were performed to assess corneal curvature (K1, K2, Km). In addition, standard logarithmic visual acuity charts were used to measure uncorrected distance visual acuity (UDVA) at 5 m and corrected distance visual acuity (CDVA). UDVA was also measured at 1 week, 1 month, and 3 months postoperatively. During the OQAS II examination, the patient’s measurements were performed with the best correction to eliminate the effect of low-order aberrations on visual performance. Since blinking may affect the tear film and thereby the scattering index, all mean OSI measurements were performed without blinking. All measurements were performed using a standardized 4 mm artificial pupil setting to ensure consistency. Examinations were conducted in a darkened room to maintain natural pupil dilation above 4 mm throughout the procedure. Subjects were instructed to blink immediately before each measurement. The examiner then promptly collected the necessary parameters to minimize potential inaccuracies resulting from fluctuations in the tear film.

### Patient questionnaire

To comprehensively assess the visual experience of patients after SMILE surgery, our center administered a standardized questionnaire 1 week postoperatively. This questionnaire focused on four key visual symptoms: watery haze, blinking frequency, visual fluctuations, and glare. A three-level scale was used to assess the frequency of these symptoms: ‘most of the time,’ ‘sometimes,’ and ‘seldom’.

### Statistical analysis

Statistical analyses were performed using IBM SPSS Statistics version 26.0 (IBM Corporation, Armonk, NY, United States). The right eye of each participant was selected for analysis. Data normality was assessed using the Kolmogorov–Smirnov test. Chi-square tests were applied for categorical variables, including sex and visual acuity (pre-operative and postoperative). Paired *t*-tests or Wilcoxon signed-rank tests were used for normally or non-normally distributed continuous variables, respectively, to analyze changes from baseline. Between-group comparisons were conducted using independent samples *t*-tests or Mann–Whitney *U*-tests, as appropriate. Correlations between variables were evaluated using Spearman’s rank correlation analysis. Statistical significance was defined as *p* < 0.05.

## Results

### Baseline comparison

In this study, we employed a continuous enrollment method to include eligible refractive surgery patients in chronological order of their visit time. We collected data from a total of 141 participants who underwent refractive surgery, analyzing their 141 right eyes. The study comprised 141 eyes from 141 patients, with 71 patients in Group A and 70 patients in Group B. Demographic data and preoperative baseline measurements for both patient cohorts are presented in Table 1. Statistical analysis revealed no significant differences between the two groups (*p* > 0.05). All surgical procedures were completed successfully, with no postoperative complications reported.

**Table 1:** Demographic data and characteristics of patients.

	Group A (*n* = 71)	Group B (*n* = 70)	*p*-value
Age (year)	23.50 ± 6.42	24.12 ± 6.17	0.899
Sex (male/female)	38/33	36/34	0.936
Pre-op S (D)	−4.89 ± 1.55	−4.69 ± 1.83	0.504
Pre-op C (D)	−0.82 ± 0.53	−0.81 ± 0.65	0.631
Pre-op SE (D)	−5.33 ± 1.31	−5.30 ± 1.23	0.537
Pre-op logMAR UDVA	1 ± 0.28	0.98 ± 0.21	0.472
Pre-op logMAR CDVA	0.02 ± 0.04	0.02 ± 0.03	0.127
Pre-op CCT (μm)	544.28 ± 35.16	546.54 ± 34.86	0.866
Km	42.78 ± 1.26	43.36 ± 1.44	0.617
OSI	0.98 ± 0.70	0.90 ± 0.54	0.994
Mean OSI	1.49 ± 0.86	1.75 ± 0.96	0.098
OV100%	1.18 ± 0.33	1.22 ± 0.32	0.435
OV20%	0.85 ± 0.31	0.87 ± 0.25	0.383
OV9%	0.53 ± 0.21	0.51 ± 0.16	0.838
MTF cutoff (c/deg)	35.54 ± 10.11	36.57 ± 9.43	0.449
SR	0.2 ± 0.06	0.2 ± 0.05	0.384
TF-OSI	0.45 ± 0.44	0.67 ± 0.67	0.377
TBUT (S)	12.51 ± 2.97	11.62 ± 3.52	0.316

Data are shown as mean ± SD (range); Group A, tear film stabilization group, TF-OSI less than 1.2; Group B, tear film instability group, TF-OSI greater than or equal to 1.2; S: spherical; C: cylinder; SE: spherical equivalent refraction; UDVA: uncorrected distance visual acuity; CDVA, corrected distance visual acuity; CCT, central corneal thickness; Km: keratometry mean; OSI, object scatter index; mean OSI, mean object scatter index; MTF cutoff: modulation transfer function cutoff; SR, Strehl ratio.OV100%, OV20%, and OV9:predicted visual acuity at contrast levels of 100, 20, and 9%;TF-OSI, tear film objective scatter index, numerically equal to Mean OSI minus OSI; TBUT, tear break-up time.

### Visual acuity outcomes and refractive outcomes

**Figure 1:**
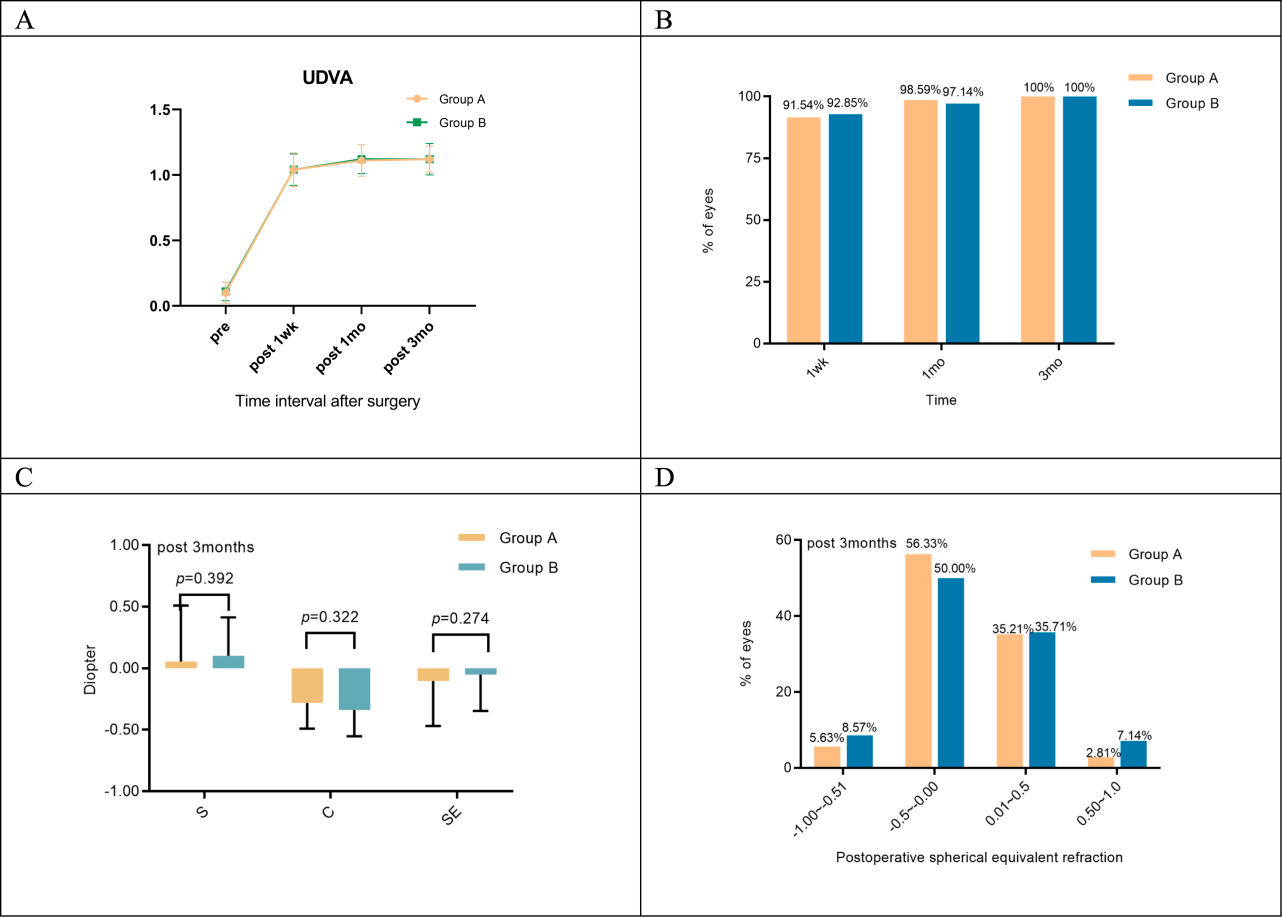
**(A)** Changes in uncorrected visual acuity after SMILE in both groups; **(B)** comparison of the proportion of UDVA ≥1.0 at different postoperative periods between the two groups; **(C)** refractive diopter after SMILE surgery in both groups; **(D)** spherical equivalent refraction.

Postoperative uncorrected distance visual acuity (UDVA) showed no statistically significant differences between Groups A and B. This consistency was observed across all follow-up periods, including 1 week, 1 month, and 3 months after surgery (*p* = 0.412, 0.563, and 0.853; Figure 1A). The proportions of patients with UDVA ≥1.0 in Groups A and B were 91.54 and 92.85% at 1 week postoperatively, 98.59 and 97.14% at 1 month postoperatively, and 100.00% in both groups at 3 months postoperatively (Figure 1B).

At 3 months post-surgery, equivalent refractive error within ±0.50 D was observed in 91.54% of patients in Group A and 85.71% of patients in Group B (Figure 1D). No significant differences were found in spherical, cylindrical, or spherical equivalent refractive errors between the two groups (*p* = 0.392, 0.322, and 0.274; Figure 1C).

### Objective optical quality

**Figure 2:**
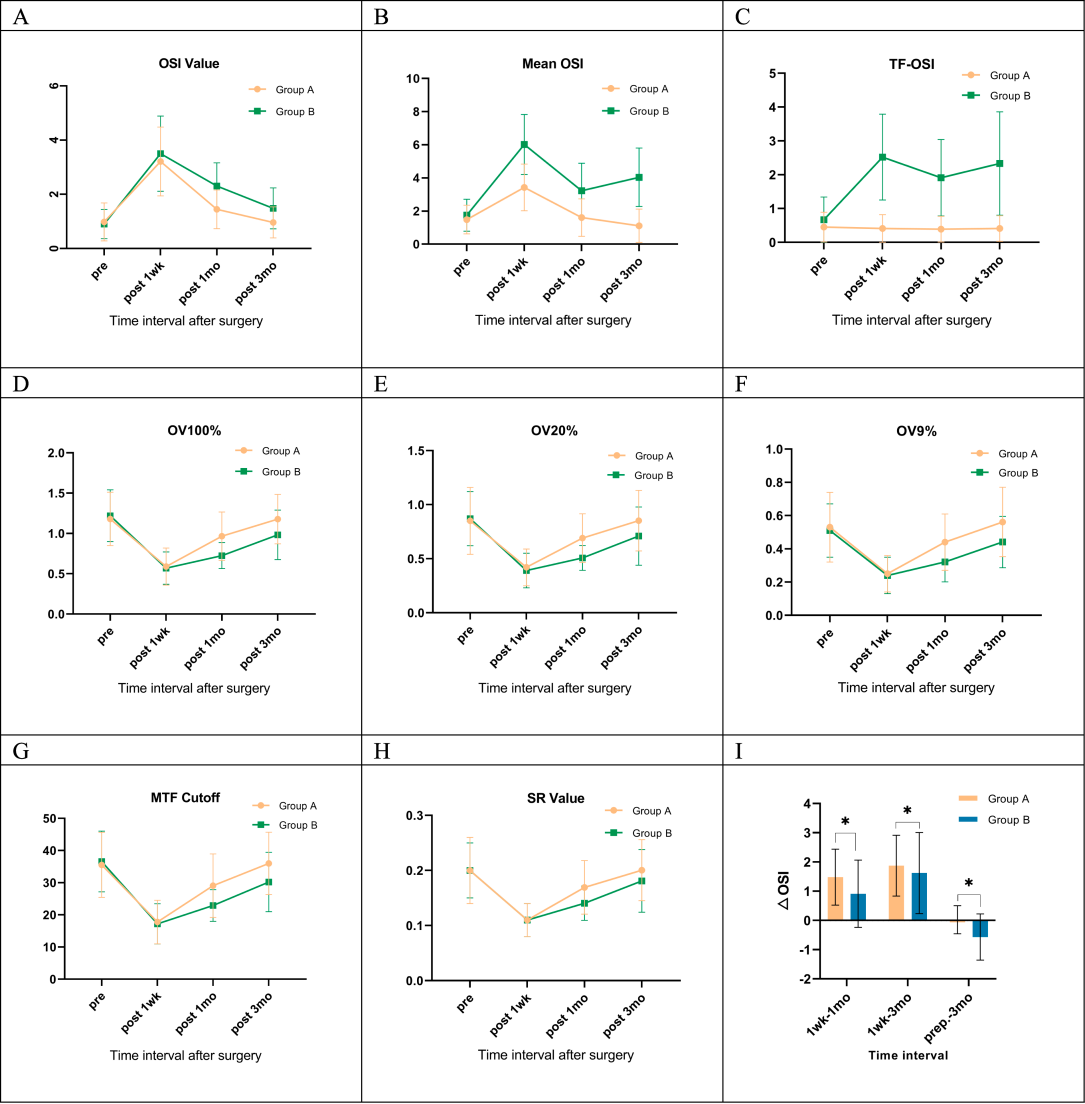
Objective visual quality parameters in both groups. **(A)** Preoperative and postoperative OSI values; **(B)** preoperative and postoperative Mean OSI values; **(C)** preoperative and postoperative TF-OSI values; **(D)** preoperative and postoperative OV100% values; **(E)** preoperative and postoperative OV20% values; **(F)** preoperative and postoperative OV9% values; **(G)** preoperative and postoperative MTF cutoff values; **(H)** preoperative and postoperative SR values; **(I)** changes in OSI values at different points in time; Group A, tear film stabilization group, TF-OSI less than 1.2; Group B, tear film instability group, TF-OSI greater than or equal to 1.2; pre: preoperative; post: postoperative; 1wk: 1 week; 1mo: 1 month; 3mo: 3 months; 1wk-1mo, OSI values at 1 week postoperatively minus 1 month postoperatively; 1wk-3mo, OSI values at 1 week postoperatively minus 3 months postoperatively; prep-3mo, preoperative OSI values minus 3 months postoperative; *p < 0.05, statistically significant.

Preoperatively and 1 week postoperatively, no significant differences were observed in most objective visual quality parameters between the two groups, with the exception of tear film-related values (Mean OSI and TF-OSI) at the 1-week postoperative assessment. However, at 1 month postoperatively, OV100%, OV20%, OV9%, MTF cutoff, and SR values were significantly higher in Group A than in Group B. In addition, OSI and Mean OSI were significantly lower in Group A than in Group B at this time point. These differences were statistically significant, and these differences persisted until 3 months postoperatively (Table 2 and Figures 2A–H, 5A,B).

**Table 2:** Comparison of objective visual quality parameters between the two groups.

Group	Pre-op	1 week	1 month	3 months	*P*(Pre-op. vs. 3 months)
OSI					
A	0.98 ± 0.70	3.21 ± 1.27^a^	1.44 ± 0.71^a^	0.96 ± 0.57	0.614
B	0.90 ± 0.54	3.50 ± 1.39^a^	2.31 ± 0.85^a^	1.48 ± 0.75	*P* < 0.05
*P*	0.994	0.328	*P* < 0.05	*P* < 0.05	
Mean OSI					
A	1.49 ± 0.86	3.43 ± 1.41^a^	1.61 ± 1.13^a^	1.11 ± 1.01	*P* < 0.05
B	1.75 ± 0.96	6.02 ± 1.81^a^	3.23 ± 1.66^a^	4.04 ± 1.76^a^	*P* < 0.05
*P*	0.098	*P* < 0.05	*P* < 0.05	*p* < 0.05	
OV100%					
A	1.18 ± 0.33	0.59 ± 0.23^a^	0.97 ± 0.30^a^	1.18 ± 0.31	0.889
B	1.22 ± 0.32	0.57 ± 0.20^a^	0.72 ± 0.16^a^	0.98 ± 0.31^a^	*P* < 0.05
*P*	0.435	0.816	*P* < 0.05	*p* < 0.05	
OV20%					
A	0.85 ± 0.31	0.42 ± 0.17^a^	0.69 ± 0.23^a^	0.85 ± 0.28	0.702
B	0.87 ± 0.25	0.39 ± 0.16^a^	0.51 ± 0.11^a^	0.71 ± 0.27^a^	0.586
*P*	0.383	0.306	*P* < 0.05	*P* < 0.05	
OV9%					
A	0.53 ± 0.21	0.25 ± 0.11^a^	0.44 ± 0.17^a^	0.56 ± 0.21	0.081
B	0.51 ± 0.16	0.24 ± 0.11^a^	0.32 ± 0.12^a^	0.44 ± 0.15^a^	*P* < 0.05
*P*	0.838	0.590	*P* < 0.05	*p* < 0.05	
MTF cutoff (c/deg)					
A	35.54 ± 10.11	17.75 ± 6.76^a^	29.08 ± 9.87^a^	36.00 ± 9.71	0.178
B	36.57 ± 9.43	17.18 ± 6.24^a^	22.90 ± 4.90^a^	30.20 ± 9.22^a^	*P* < 0.05
*P*	0.449	0.671	*P* < 0.05	*p* < 0.05	
SR					
A	0.20 ± 0.06	0.11 ± 0.03^a^	0.17 ± 0.05^a^	0.20 ± 0.06	0.497
B	0.20 ± 0.05	0.11 ± 0.03^a^	0.14 ± 0.03^a^	0.18 ± 0.06	0.064
*P*	0.671	0.173	*p* < 0.05	0.051	
TF-OSI					
A	0.45 ± 0.44	0.41 ± 0.41	0.39 ± 0.38	0.41 ± 0.38	0.692
B	0.67 ± 0.67	2.52 ± 1.27^a^	1.91 ± 1.13^a^	2.33 ± 1.53^a^	*P* < 0.05
*P*	0.377	*P* < 0.05	*P* < 0.05	*P* < 0.05	

Data are shown as mean ± SD (range); Group A, tear film stabilization group, TF-OSI less than 1.2; Group B, tear film instability group, TF-OSI greater than or equal to 1.2; OSI, object scatter index; Mean OSI, mean object scatter index; MTF cutoff: modulation transfer function cutoff; SR, Strehl ratio.OV100%, OV20%, and OV9: predicted visual acuity at contrasts levels of 100, 20, and 9%; TF-OSI, tear film objective scatter index, numerically equal to Mean OSI minus OSI. The a in the corner label indicates a statistically significant difference from the preoperative value.

Both groups demonstrated elevated Objective Scatter Index (OSI) and Mean OSI values compared to their preoperative baselines at both 1 week and 1 month post-surgery. Concurrently, there were decreases in OV100%, OV20%, OV9%, MTF cutoff, and SR values (Table 2). At 3 months postoperatively, no significant changes were observed in objective visual quality parameters in Group A compared to preoperative levels. However, in Group B, all values related to objective visual quality showed significant differences compared to their preoperative values, except for the SR value (*p* < 0.05, Figures 2A–D, 5C). Group A exhibited more pronounced changes in OSI than in Group B. This trend was observed during two distinct intervals: from 1 week to 1 month postoperatively, and from 1 month to 3 months after the procedure (Figure 2I). Furthermore, Group A exhibited no statistically significant changes in TF-OSI across all postoperative evaluations. In contrast, Group B demonstrated a notable increase in TF-OSI at each postoperative assessment compared to preoperative measurements, and these elevations in Group B were found to be statistically significant (*p* > 0.05, Table 2 and Figures 2C, 5D). The two groups showed differences in TBUT one week after surgery (Figure 4A). Pre- and postoperative TF-OSI showed a moderate negative correlation with TBUT in both groups (Figure 4A).

### Comparison of OQAS II and subjective visual acuity measures

**Figure 3:**
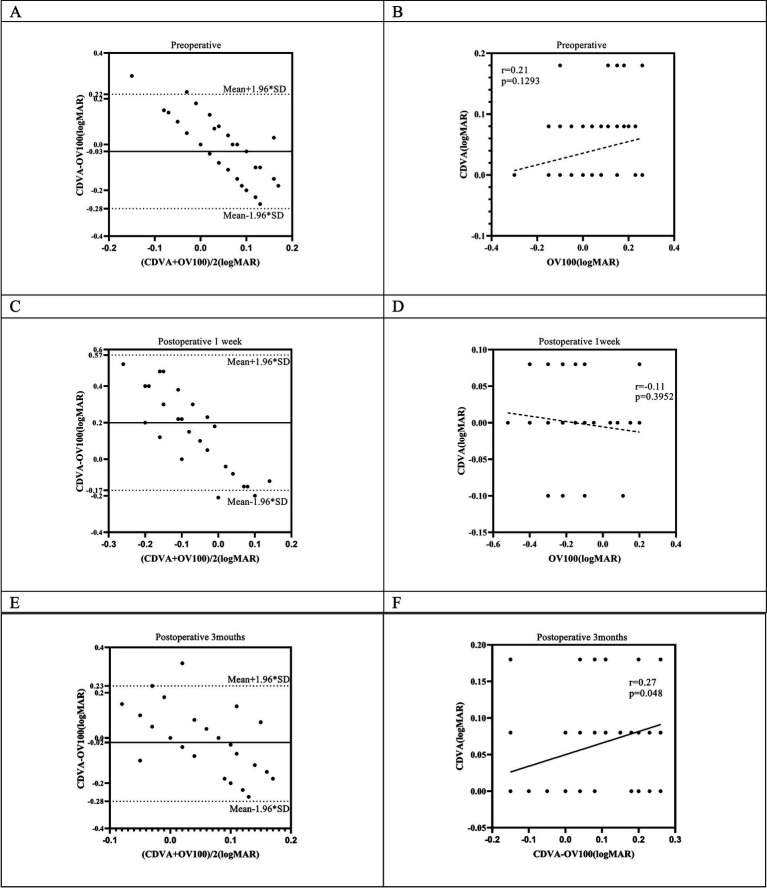
Agreement analysis of Bland–Altman plots **(A,C,E)** and correlations **(B,D,F)** of subjective visual acuity with predicted visual acuity at preoperative, 1 week postoperative, and 3 months postoperative, respectively.

The study selected the tear film stabilization group to analyze the relationship between OV100% and corrected distance visual acuity (CDVA). The results showed that OV100%, as calculated by OQAS II, significantly underestimated CDVA preoperatively by −0.04 logMAR (*p* = 0.043) and significantly overestimated CDVA by 0.25 logMAR at 1 week postoperatively (*p* < 0.001). At 3 months postoperatively, the difference between the two means was not statistically significant (*p* = 0.112) (Table 3). The distribution of OV100% data, as measured by the OQAS II at various pre- and postoperative intervals, consistently exhibited larger standard deviations compared to those obtained through subjective assessment methods. Bland–Altman plots were used to analyze the agreement, showing the mean differences as described. The deviation between the methods was more pronounced at 1 week after SMILE but regressed to preoperative levels at 3 months postoperatively. The plots showed a weak correlation between the two methods at all time points, with a slightly stronger correlation observed at 3 months postoperatively (Figures 3A–F).

**Table 3:** Visual acuity obtained by subjective measurements and objective measurements with the OQAS II at 100% contrast at different time points.

	Subjective Mean ± SD	Objective Mean ± SD	*P*-value
Preoperative	0.02 ± 0.04	0.06 ± 0.15	0.043
1 week	0.03 ± 0.05	−0.22 ± 014	*p* < 0.001
3 months	0.04 ± 0.05	0.06 ± 0.11	0.112

### Patient questionnaire result

The results of the patient satisfaction survey are shown in Figures 6A–D. The analysis revealed significant differences between the subgroups in terms of individual subjective feelings. A higher percentage of patients in Group B reported increased blinking frequency and visual fluctuations than in Group A. Significantly more patients in Groups A1 and B1 reported foggy vision and halos than in Groups A2 and B2 (Figures 6A–D).

**Figure 4:**
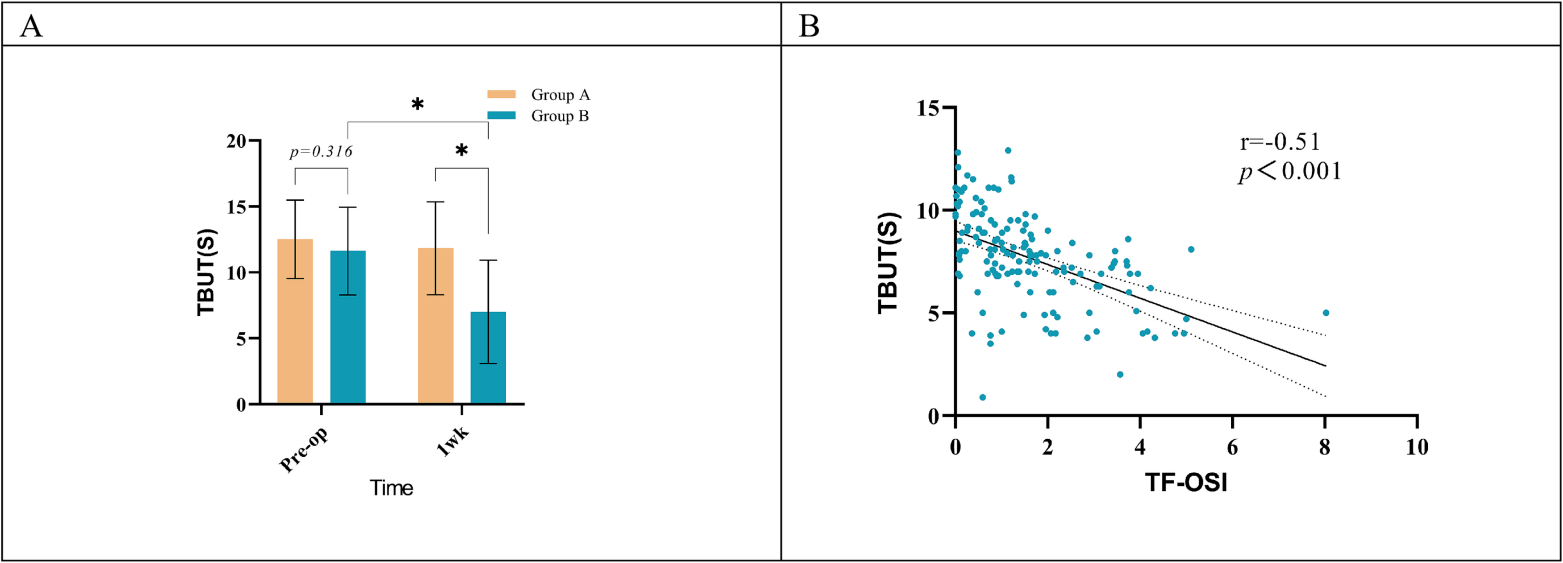
**(A)** Comparison of 1 week postoperative and preoperative TBUT within both groups; **(B)** correlation of TBUT and TF-OSI in all groups. *Implies a statistical difference between the two groups, *p* < 0.05.

**Figure 5:**
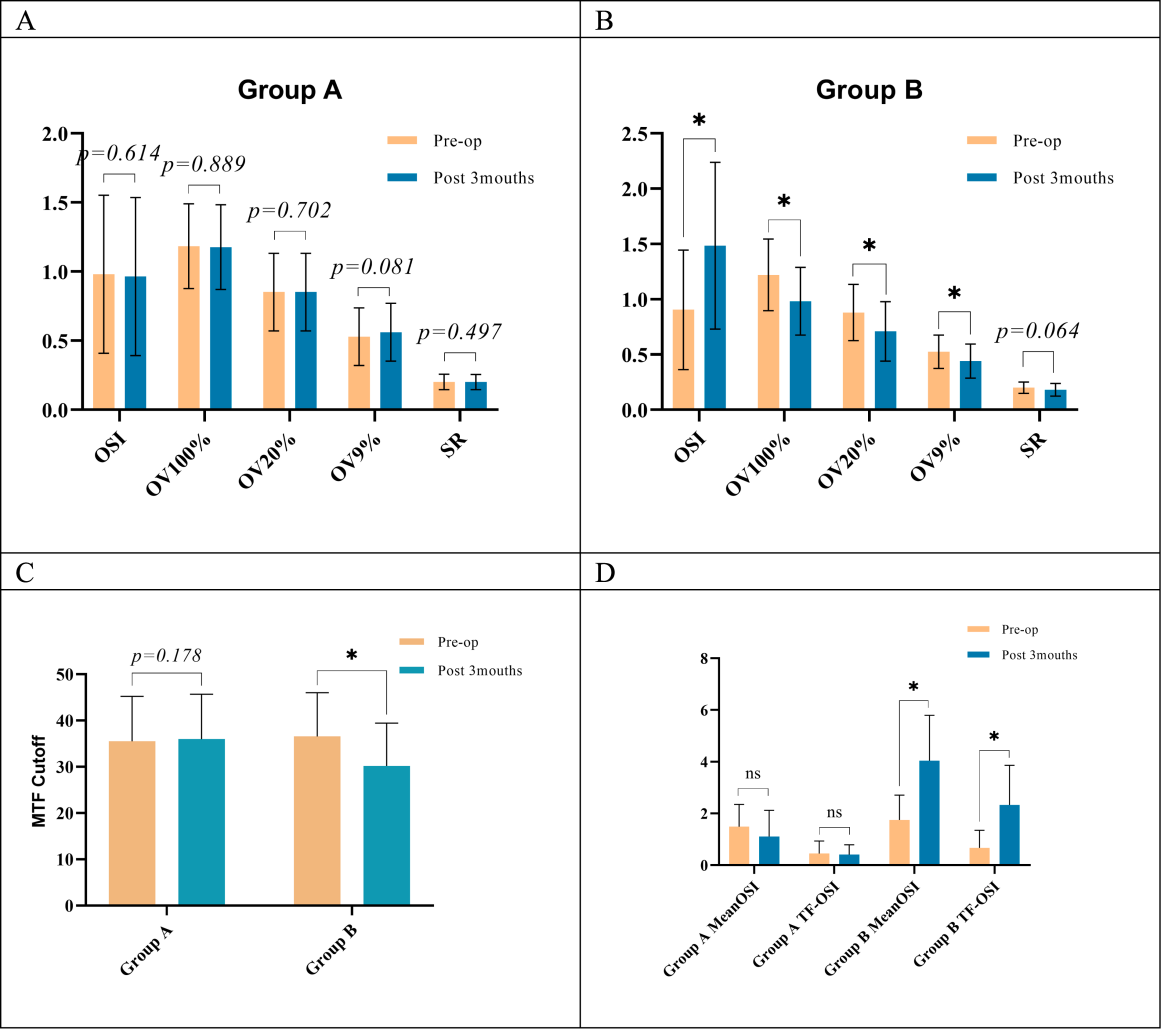
**(A)** Comparison of 3-month postoperative and preoperative objective visual quality indicators within group A; **(B)** comparison of 3-month postoperative and preoperative objective visual quality indicators within group B; **(C)** comparison of 3-month postoperative and preoperative MTF cutoff indicators within both groups; **(D)**, comparison of Mean OSI and TF-OSI at 3 months postoperatively versus preoperatively within both groups; pre: preoperative; post: postoperative; OSI, object scatter index; Mean OSI, mean object scatter index; MTF cutoff: modulation transfer function cutoff; SR, Strehl ratio. OV100%, OV20%, and OV9: predicted visual acuity at contrast levels of 100, 20, and 9%; TF-OSI, tear film objective scatter index, numerically equal to Mean OSI minus OSI. *Implies a statistical difference between the two groups, *p* < 0.05.

**Figure 6:**
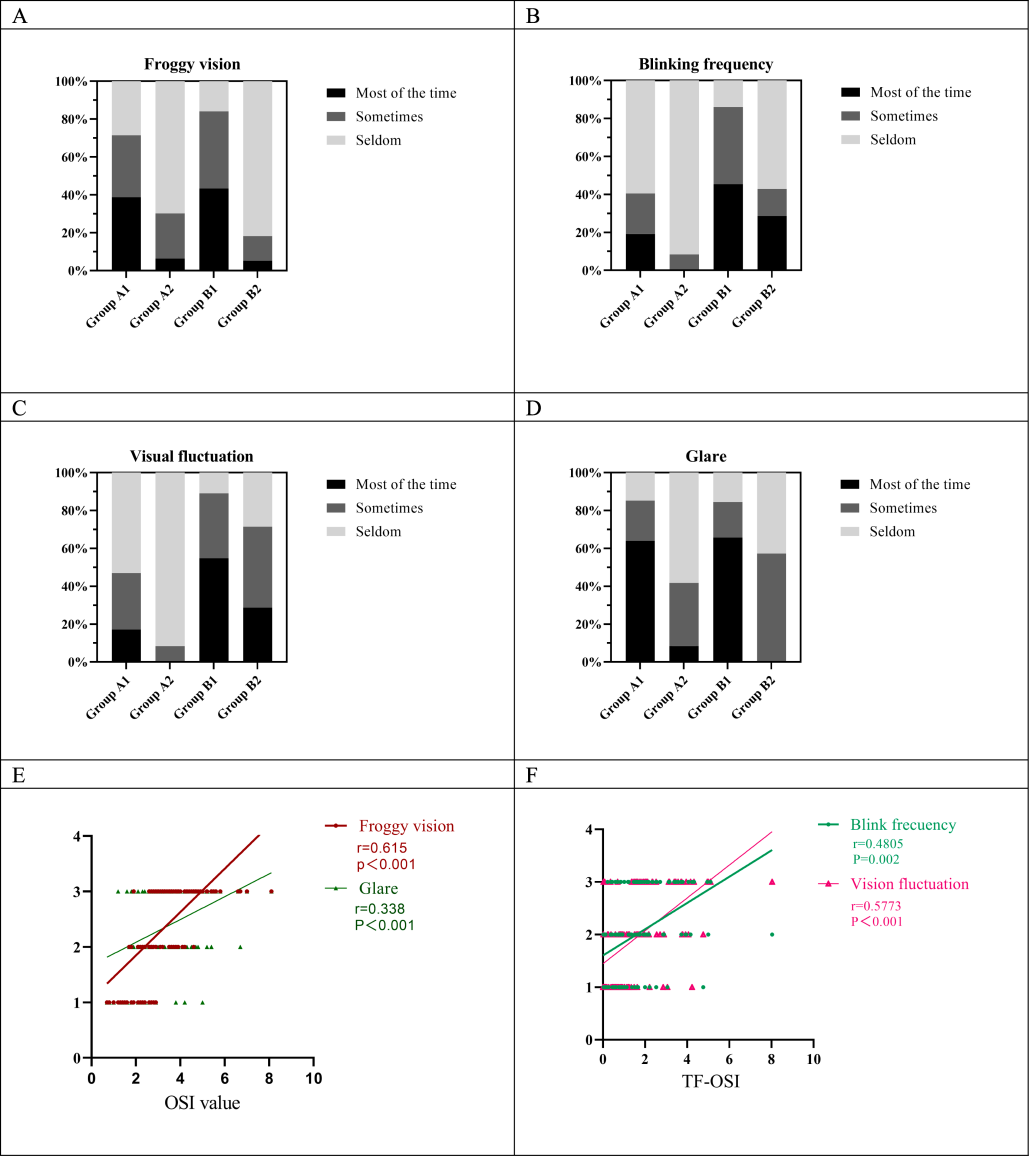
Postoperative responses of subgroups to questionnaire items; **(E,F)** correlation of each symptom with OSI and TF-OSI.

Correlation analysis revealed significant relationships between objective measures and patient-reported symptoms. OSI values positively correlated with patient-reported foggy vision (*r* = 0.615, *p* < 0.001) and glare (*r* = 0.338, *p* < 0.001). In addition, TF-OSI values showed moderate positive correlations with blink frequency (*r* = 0.481, *p* = 0.002) and fluctuating visual acuity (*r* = 0.577, *p* < 0.001) (Figures 6E,F).

## Discussion

Previous studies have demonstrated that tear film instability leads to increased optical aberrations (13, 14) and enhanced light scattering (10). These factors contribute to fluctuations in visual quality, a common complaint among patients with dry eye disease (15). Patients with dry eye disease have reported more frequent fluctuations in visual acuity and an increased blinking rate (16, 17), which aligns with our findings. Corneal refractive surgery has been identified as a risk factor for dry eye disease (18). Post-surgical dry eye may delay corneal healing and increase the risk of refractive regression (19). The interplay between tear film instability, optical aberrations, and visual quality fluctuations highlights the importance of maintaining ocular surface health in both pre-operative and postoperative patients. Research indicates that OSI values typically rise after SMILE procedures but tend to revert to their initial levels within a quarter-year following surgery (20, 21). The objective of this study was to conduct a comparative analysis of visual quality recovery in patients with varying degrees of tear film stability during the early postoperative period, using objective measurements. This investigation has two primary purposes. First, it provides a more comprehensive understanding of how tear film stability influences postoperative recovery. Second, it offers valuable reference information for clinical practice in refractive surgery. By examining the relationship between tear film stability and visual outcomes following SMILE, we aim to enhance our ability to predict and manage postoperative visual quality fluctuations. These findings could help improve patient selection criteria and postoperative care protocols in refractive surgery.

Visual acuity and refraction are key indicators of the efficacy of corneal refractive surgery (22). Previous studies have suggested that postoperative dry eye may lead to refractive regression (19, 23). However, patients in both groups demonstrated significant improvement in uncorrected visual acuity postoperatively. Analysis of early postoperative outcomes (at 1 week, 1 month, and 3 months) showed comparable results among all groups in terms of uncorrected vision and refractive measurements. No statistically significant variations were detected during these initial follow-up periods, and no significant refractive regression was observed. These findings diverge from those of some previous studies (24). Several factors may account for this discrepancy: First, the degree of tear film instability in our cohort may not have been sufficient to induce significant refractive regression. Second, continuous improvements in refractive surgical techniques may have reduced the incidence and severity of postoperative dry eye, thereby mitigating the risk of refractive regression. In addition, the relatively short follow-up period in our study may have precluded the observation of long-term refractive changes. To validate these results, longer-term follow-up studies are needed, which would offer a more comprehensive understanding of the relationship between tear film stability, refractive outcomes, and visual acuity after corneal refractive surgery.

Our study revealed that OSI and Mean OSI values were significantly higher at both 1 week and 1 month after surgery in both groups compared to the preoperative levels. This finding aligns with previous studies suggesting that refractive surgery can lead to a temporary increase in OSI (25). Several factors may contribute to increased postoperative OSI, including corneal surface irregularities, eccentricity, tear film instability, dry eye symptoms, and lens removal techniques (26, 27). The majority of studies indicate that OSI typically returns to preoperative levels 3 months post-surgery (20). This transient increase in OSI usually improves gradually during the postoperative recovery process. In our study, the tear film stabilization group (Group A) showed 3-month postoperative OSI values similar to preoperative levels, which is consistent with previous findings. However, the tear film unstable group (Group B) showed significantly different OSI values compared to preoperative levels at 3 months postoperatively, which warrants further investigation. While both groups showed no significant difference in OSI at 1 week postoperatively, Group A exhibited significantly lower scatter than Group B at both 1 and 3 months postoperatively. OSI recovery was also greater in the tear film stabilization group at these recovery time points of 1 week to 1 month and 1 month to 3 months. This phenomenon may reflect the crucial influence of tear film stability on postoperative visual quality recovery. We hypothesize that the early postoperative increase in OSI (within 1 week) primarily results from the surgery itself, with minimal influence from tear film status. Over time, tear film stability gradually becomes a key factor affecting OSI recovery (24). MTF cutoff is a measure of the eye’s ability to resolve details. A higher MTF cutoff value indicates better visual acuity, particularly in peripheral vision. Similarly, a higher Strehl ratio (SR) signifies better optical quality and fewer aberrations in the visual system (28). At 3 months postoperatively, there was no significant difference between group A and its preoperative levels. However, in group B, both MTF cutoff and SR values were lower compared to preoperative levels, with a statistically significant decrease in the MTF cutoff value. This suggests that the resolution capability and optical quality of eyes in group B had not returned to preoperative levels 3 months after surgery. Tear film instability may lead to delayed OSI recovery, explaining why Group B had not fully returned to preoperative levels at 3 months postoperatively. Patients in Group B exhibited tear film instability 1 week postoperatively. We speculate that these individuals may have had a pre-existing asymptomatic subclinical dry eye condition. Although their tear film-related parameters such as TF-OSI and TBUT did not show statistically significant differences from Group A, the overall mean values were slightly less favorable. The surgical intervention likely disrupted the ocular surface balance, triggering tear film instability (29). The present study indicates a moderately strong negative correlation between TF-OSI and TBUT, rather than a very strong negative correlation, which aligns with the findings of Yu Chen et al. (12). This discrepancy may be attributed to our methodological approach. In our study, the OSI was measured using OQAS II with a 4-mm artificial pupil, while TBUT was determined by the appearance of the first dry spot on the cornea. Notably, many patients exhibited dry spots outside the pupillary area, which could contribute to greater variability in scattering measurements. This finding underscores the importance of postoperative tear film management, particularly in patients with preexisting tear film instability. These observations deepen our understanding of visual quality recovery following SMILE surgery and lay a crucial foundation for developing individualized postoperative management strategies. Future studies should explore methods to improve tear film stability, potentially promoting faster postoperative visual quality recovery.

Visual acuity assessment is a crucial indicator of ophthalmic surgical outcomes. Previous studies have primarily used predicted visual acuity to assess optical quality (30–32) and explored its agreement with subjective visual acuity (33–35). However, to date, no study has specifically assessed the agreement between short-term predicted visual acuity and subjective visual acuity after SMILE surgery. Higher limits of agreement (LoA) and low correlations typically suggest that one measure cannot directly substitute for another (36), which is a crucial consideration when evaluating post-SMILE visual acuity outcomes. To facilitate comparison with existing literature, we converted OV100% and CDVA from decimal to logarithmic scales using Khoshnood et al.’s (37) method. Our preoperative and postoperative results align with studies reporting similar outcomes after phakic IOL implantation (0 logMAR) (30) and cataract surgery with monofocal IOL (−0.02 and 0 logMAR) (38). In addition, some patients in our 1-week postoperative study exhibited a higher bias, similar to results observed after cataract surgery with dual-focus intraocular implants (−0.18 logMAR) (31). The precise mechanism underlying these deviations remains unclear, but in healthy subjects, visual acuity is primarily limited by neurological factors, which could lead to OV100% overestimation preoperatively and at 3 months postoperatively (39). Our study revealed weak or no correlation between CDVA and OV100% measurements, with larger LoAs observed preoperatively, 1 week, and 3 months after SMILE surgery. This finding suggests that the OQAS II system alone cannot accurately predict subjective visual acuity, which is consistent with the findings of Chen et al. (40), particularly 1 week after SMILE surgery when the bias was more pronounced.

To date, few studies have examined the relationship between clinical parameters and patients’ subjective visual symptoms following SMILE (41, 42). In this study, we identified significant differences in visual performance among patient subgroups 1 week post-SMILE using a questionnaire. Further analysis of subjective visual perception revealed a moderate correlation between OSI values and the severity of watery haze and glare. Patients with higher OSI values reported more frequent watery haze and glare experiences. OSI, as a scattering index, reflects the transparency and uniformity of the refractive medium (43). In SMILE surgery, which involves only corneal tissue, changes in postoperative OSI values likely stem from corneal alterations. The association between higher OSI values and increased watery haze and glare may result from enhanced light scattering caused by corneal surface irregularities or postoperative inflammation. In addition, patients with higher TF-OSI reported more visual fluctuations and increased blinking. This finding highlights the significant influence of tear film stability on postoperative visual quality. SMILE surgery may temporarily affect tear film distribution and stability, resulting in elevated TF-OSI.

This study provides valuable findings but has several limitations. The limited sample size may have introduced bias, affecting the generalizability of the results. The study primarily focused on short-term postoperative outcomes, which may not fully capture the long-term impact of tear film stability on visual quality. We analyzed subjective visual quality and tear break-up time (TBUT) only at 1 week postoperatively, which limits our ability to assess changes over multiple time points. To address these limitations and assess the long-term impact of tear film stability on visual quality and surgical outcomes, future research should extend the follow-up period. Increasing the sample size would improve statistical power and reduce bias. Adding more items to the questionnaire would provide more comprehensive data. Analyzing changes in relevant parameters at multiple postoperative time points would provide a better understanding of the relationship between tear film stability and postoperative visual outcomes. These improvements would provide a more comprehensive assessment of the impact of tear film stability on visual quality and overall surgical outcomes.

## Conclusion

In conclusion, this study shows that poor tear film stability negatively impacts objective visual quality recovery and prolongs corneal healing time following SMILE surgery. Patients with unstable tear films require longer recovery. Tear film instability does not cause refractive regression in the short term (3 months post-surgery). The study shows that OV 100% is not a highly accurate predictor of visual acuity, especially 1 week postoperatively. A clear correlation exists between objective visual quality and subjective perception. The OQAS II system is an effective tool for assessing tear film stability and objective visual quality after refractive surgery.

## Data Availability

The raw data supporting the conclusions of this article will be made available by the authors, without undue reservation.
